# Affordable Three-Dimensional Printed Heart Models

**DOI:** 10.3389/fcvm.2021.642011

**Published:** 2021-06-04

**Authors:** Gorka Gómez-Ciriza, Tomás Gómez-Cía, José Antonio Rivas-González, Mari Nieves Velasco Forte, Israel Valverde

**Affiliations:** ^1^Fabrication Laboratory, Virgen del Rocio University Hospital, Institute of Biomedicine of Seville (IBIS), Seville, Spain; ^2^Plastic Surgery and Burns Unit, Virgen del Rocio University Hospital, Seville, Spain; ^3^Division of Imaging Sciences and Biomedical Engineering, King's College London, The Rayne Institute, St. Thomas' Hospital, London, United Kingdom; ^4^Cardiovascular Pathology Unit, Institute of Biomedicine of Seville (IBIS), Centro de Investigación Biomédica en Red Enfermedades Cardiovasculares, Hospital Virgen de Rocio/Consejo Superior de Investigaciones Científicas/University of Seville, Seville, Spain; ^5^Department of Congenital Heart Disease, Evelina London Children's Hospital, Guy's and St. Thomas' NHS Foundation Trust, London, United Kingdom

**Keywords:** affordable 3d printing, surgical planning, interventional planning, heart models, medical education

## Abstract

This is a 7-years single institution study on low-cost cardiac three-dimensional (3D) printing based on the use of free open-source programs and affordable printers and materials. The process of 3D printing is based on several steps (image acquisition, segmentation, mesh optimization, slicing, and three-dimensional printing). The necessary technology and the processes to set up an affordable three-dimensional printing laboratory are hereby described in detail. Their impact on surgical and interventional planning, medical training, communication with patients and relatives, patients' perception on care, and new cardiac device development was analyzed. A total of 138 low-cost heart models were designed and printed from 2013 to 2020. All of them were from different congenital heart disease patients. The average time for segmentation and design of the hearts was 136 min; the average time for printing and cleaning the models was 13.5 h. The average production cost of the models was €85.7 per model. This is the most extensive series of 3D printed cardiac models published to date. In this study, the possibility of manufacturing three-dimensional printed heart models in a low-cost facility fulfilling the highest requirements from a technical and clinical point of view is demonstrated.

## Introduction

Personalized three-dimensional (3D) printed models have become a powerful tool for clinicians and surgeons in recent years. 3D printed models provide tactile feedback and enable users to simulate realistic anatomical navigation. All these operations are difficult to reproduce in computer software. 3D replicas offer an enhanced appreciation of the visuospatial relationship between anatomical structures (1). This can be translated into shorter operative time, reduced exposure to general anesthesia, shorter wound exposure time, and reduced intraoperative blood loss (2).

In recent years, researchers and clinicians have demonstrated that patient-specific heart models of different pathologies are useful to plan surgical and interventional procedures and also to teach students and communicate with patients and their families (1, 3–10). Different clinical areas have incorporated this technology in their current clinical practice including plastic (11), maxillofacial (12), orthopedic (13), or cardiac surgery (6).

Although many publications describe how useful 3D models are in different medical applications (14), one of the main issues of this technology is the high cost inherent to the equipment and software needed to start a 3D printing facility (15). More efforts are necessary to maximize the benefits of this solution while reducing the costs associated (16–18).

3D printing is a process that requires several complex steps, and it is necessary to study and analyze the investment needed for each one. The price of the software utilized for 3D printing varies from 0 to more than €10,000 per license and year. 3D printers can be purchased following a wide range of prices, ranging from a few hundred euros to more than €150,000. Materials also vary from €15/kg to more than €300/kg. All these expenses associated with the design and fabrication of the models are key factors that can complicate the translation of this technology from research to normal clinical practice.

The design and fabrication of a patient-specific 3D printed heart model has been previously described in the literature (3, 5, 19–21). In this article, we present our institutional experience using affordable technology over the last 7 years in the field of cardiac 3D printing, analyzing and describing each technical and economic factor involved in the design and manufacturing process. The aim of this article is to demonstrate the feasibility from a technical and economic point of view. The clinical utility of the models is briefly described in this article to contextualize the application of the technology.

### Standard Protocol for Medical 3D Printing (State of the Art)

#### Segmentation

Medical images from CT (computed tomography), CMR (cardiac magnetic resonance), or echocardiography are stored in the digital imaging and communication in medicine (DICOM) format and loaded into segmentation software.

There are several tools for segmentation; some of them are free and open source, and others are licensed. Mimics (Materialize NV, Leuven, Belgium) is a payment license program included in the Mimics Innovation Suite (~€10,000/license and years) and one of the most cited programs in scientific literature (22). Osirix (Pixmeo, Geneva, Switzerland) only runs on Mac; it has a free version and an FDA- and CE-approved version that costs €756 per year and license.

There are other popular free open-source alternatives in medical 3D printing such as MITK (German Cancer Research Center, Heidelberg, Germany), ITK Snap, and 3D Slicer.

All of them have similar reliability in the volume segmentation of the anatomy and similar accuracies (23). Image resolution under 1 mm is recommended to perform high-quality reconstructions (24). Some programs are able to perform a semi-automated segmentation, but it is mainly reliable on normal heart anatomies. For complex congenital heart disease, a manual segmentation is recommended. However, it can be a laborious task and user-dependent as it relies on the expertise on heart morphology and image processing of the person performing the segmentation (22).

#### Mesh Optimization

Once the segmentation is finished, the mesh is exported as an STL file (standard tessellation language). The meshes directly obtained from the segmentation are not valid for 3D printing, and it is necessary to perform some modifications to the geometries. There is often noise derived from image artifacts or related to the lack of contrast in venous areas. When the image study has low spatial resolution, the meshes have irregular surfaces that do not accurately represent the true anatomy of the patient. All this inaccuracies have to be corrected in order to obtain a reliable replica of the anatomy.

Some of the most popular computer-aided design (CAD) programs have a yearly license fee. However, there is an increasing amount of options that are available for free. Some examples of free open-source software are as follows: Meshmixer (Autodesk Inc., San Rafael, CA, USA) and Blender (Blender Foundation, Amsterdam, Netherlands). Some licensed software packages are SketchUp (Trimble Inc, Sunnyvale, CA, USA) with research and professional versions ($49 and $695), SolidWorks (Solidworks, Dassault Systèmes, Waltham, MA, USA) from $150 to $8,000, ZBrush 4R8 (Pixologic, Los Angeles, CA, USA) $795, Rhinoceros (Mac Neel Europe, Barcelona, Spain) costing €195 to €1,695, and 3Matic (Materialize NV, Leuven, Belgium) included in the Mimics Innovation Suite (€10,000/license and year).

#### Slicing

The last step of the process is the lamination or slicing. Some printers have its own programs like Objet Studio (Stratasys Ltd., Eden Prairie, MN, USA) for Stratasys printers, Z-Suite (Zortrax, Olsztyn, Poland) for Zortrax printers, PreForm (Formlabs, Somerville, MA, USA), and MakerBot Print (MakerBot Industries, LLC, Brooklyn, NY, USA) for MakerBot. Others can work with open-source software such as Cura (Ultimaker BV, Geldermalsen, Netherlands), Repetier (Hot-World, Willich, Germany), Slic3r, or KiSSlicer.

Prior to slicing, it is necessary to define the parameters of the printing process. Once finished, the 3D mesh is laminated in different layers and transferred to the 3D printer.

#### 3D Printing Technologies

All 3D printing technologies are based on the same process, deposition of successive layers of material to create an object. The most relevant technologies used in medical field are Stereolithography, Polyjet, and Fused Deposition Modeling. The most important characteristics of each of these methods are summarized in Table 1.

**Table 1:** Advantages and drawbacks of the most common 3D printing techniques.

**Technique**	**Advantages**	**Disadvantages**
Stereolithography (SLA)	High precision Medical-grade materials	Moderate strength Small size models at low cost Expensive materials
Polyjet	High precision Medical-grade materials Very flexible models	Low strength and temperature resistance High cost Low durability
Fused Deposition Modeling (FDM)	Low cost Large size High resistance	Low speed Low reliability Medium flexibility of models

*Stereolithography (SLA)* is based on the photopolimerization of epoxy resin layers with an ultraviolet (UV) light. Models need support structures to be printed; these supports are removed manually.

- *Advantages*: The precision of the pieces manufactured can reach 0.025 mm, and it is possible to make internal cavities and complex structures.
- *Disadvantages*: It is more expensive than other technologies because of the high cost of the printing materials and the maintenance required. Low-cost printers have a limited printing size. Max dimensions in Form2 (FormLabs Inc, Somerville, MA, USA) are 145 × 145 × 175 mm. The printer is affordable (€3,800), but flexible materials are more expensive (€190/L).

*Polyjet* (Stratasys Ltd., Eden Prairie, MN, USA) photopolymers are deposited in a wide area, and it is possible to print in different materials at the same time.

- *Advantages:* The support is dissolvable and washable in a subsequent step. The quality of the printing is similar to that of SLA. Flexible materials (Agilus and Tango) are soft and emulate the stiffness of cardiac tissues.
- *Disadvantages:* The maintenance required is considerable. Machines and materials are also expensive, and there are no low-cost options in the market (their price ranges from €30,000 to more than €150,000). A significant amount of support material is needed for each model, increasing the total cost (25). Flexible materials in Polyjet technology can break without much difficulty as they get easily torn. The cost is over €300/L.

*Fused deposition modeling (FDM)* is based on a continuous extrusion of a melted filament from a temperature-controlled nozzle. FDM is the most affordable and most commonly used technology for medical 3D printing.

The model is made by solidification and adhesion of fine lines of thermoplastic material to form the layers.

The most important FDM 3D printer machines are the following: Prusa i3 MK3S (Prusa Research, Prague, Czechia), LulzBot TAZ 6 (Aleph Objects, Inc., Loveland, CO, USA), and Ultimaker 3 (Ultimaker BV, Geldermasen, Netherlands).

- *Advantages:* There are lots of printer models under €4,000, and they are able to print with Z-axis precision under 0.05 mm. The majority of FDM 3D printers accept several rigid and flexible materials.
- *Disadvantages:* FDM low-cost printers have lower resolution, speed, and reliability than the other technologies. The flexibility of the models printed with flexible filaments in FDM printers is inferior to models printed in Polyjet printers.

## Methods

### Patient Recruitment

Patients with congenital heart disease were recruited from several hospitals in Spain, United Kingdom, Netherlands, Italy, Germany, Lebanon, and Canada. Imaging datasets were transferred to our secure server. All 3D printing models were segmented and printed in our unit.

### Image Acquisition and Segmentation

3D models were created from computed tomography (CT) or cardiac magnetic resonance (CMR).

The preference between one imaging technique or another was based on an institutional protocol, the main anatomical structure of interest to be visualized, and the age of the patient, with a tendency to use CMR in younger population to avoid radiation (26). 3D echocardiography was also utilized in combination with CT or CMR when cardiac valves were essential for the segmentation (27, 28).

**Figure 1:**
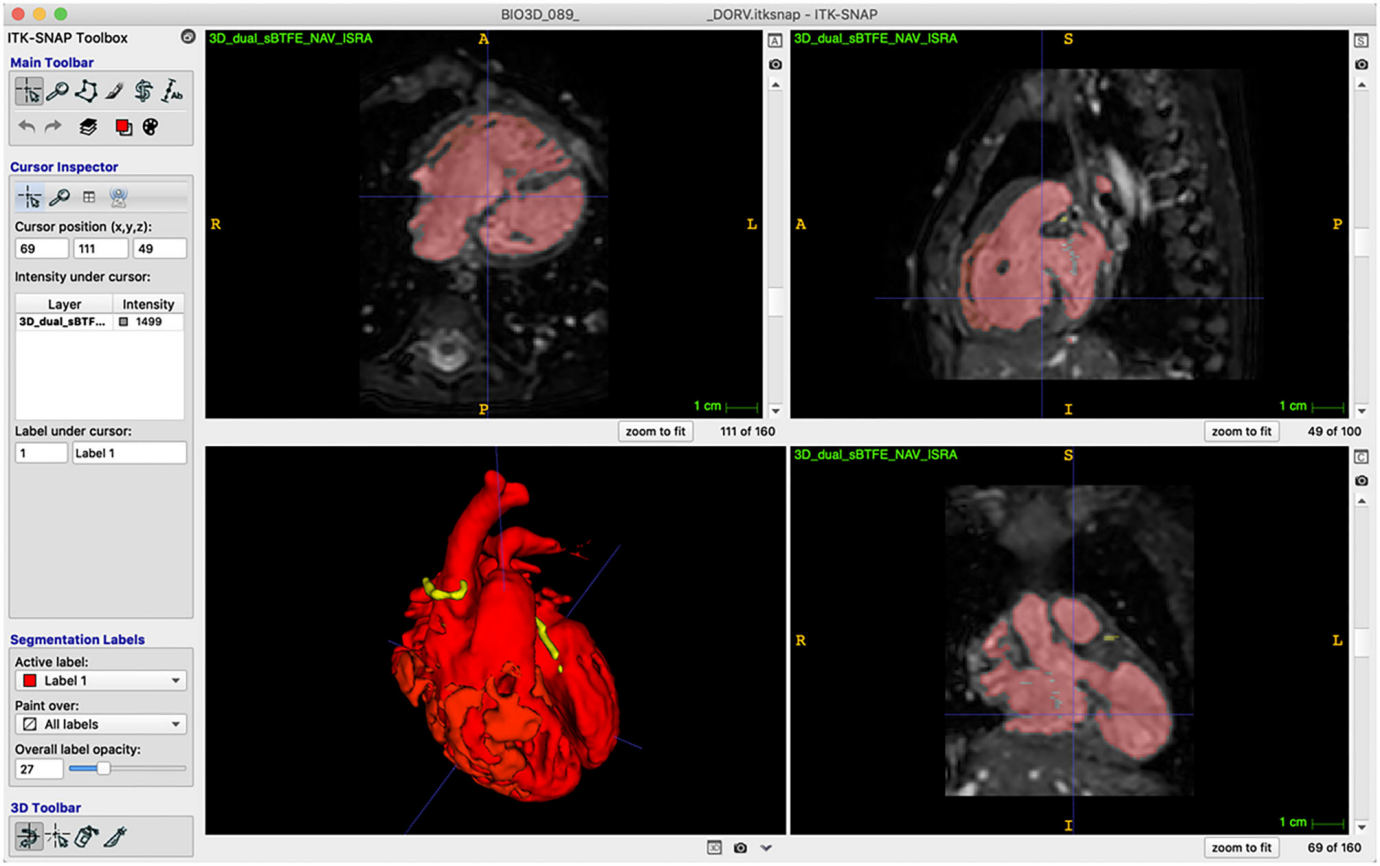
ITK Snap Segmentation. ITK toolbox on the left side of the screen. Axial (up left), sagittal (up right), 3D reconstruction (down left), and coronal (down right) views in ITK-Snap. Thresholding and manual segmentation were applied to select blood pool (red) and coronary arteries (yellow).

Free open software ITK-SNAP was the program selected for segmentation (Figure 1). Different algorithms were used, such as thresholding, classification preset, clustering, and edge attraction. After the upper and lower thresholding levels were set, a number of seeds were applied. Seeds were grown using reiterative steps. When the growing seeds collided, the affected anatomical structures were manually separated.

### Mesh Optimization

**Figure 2:**
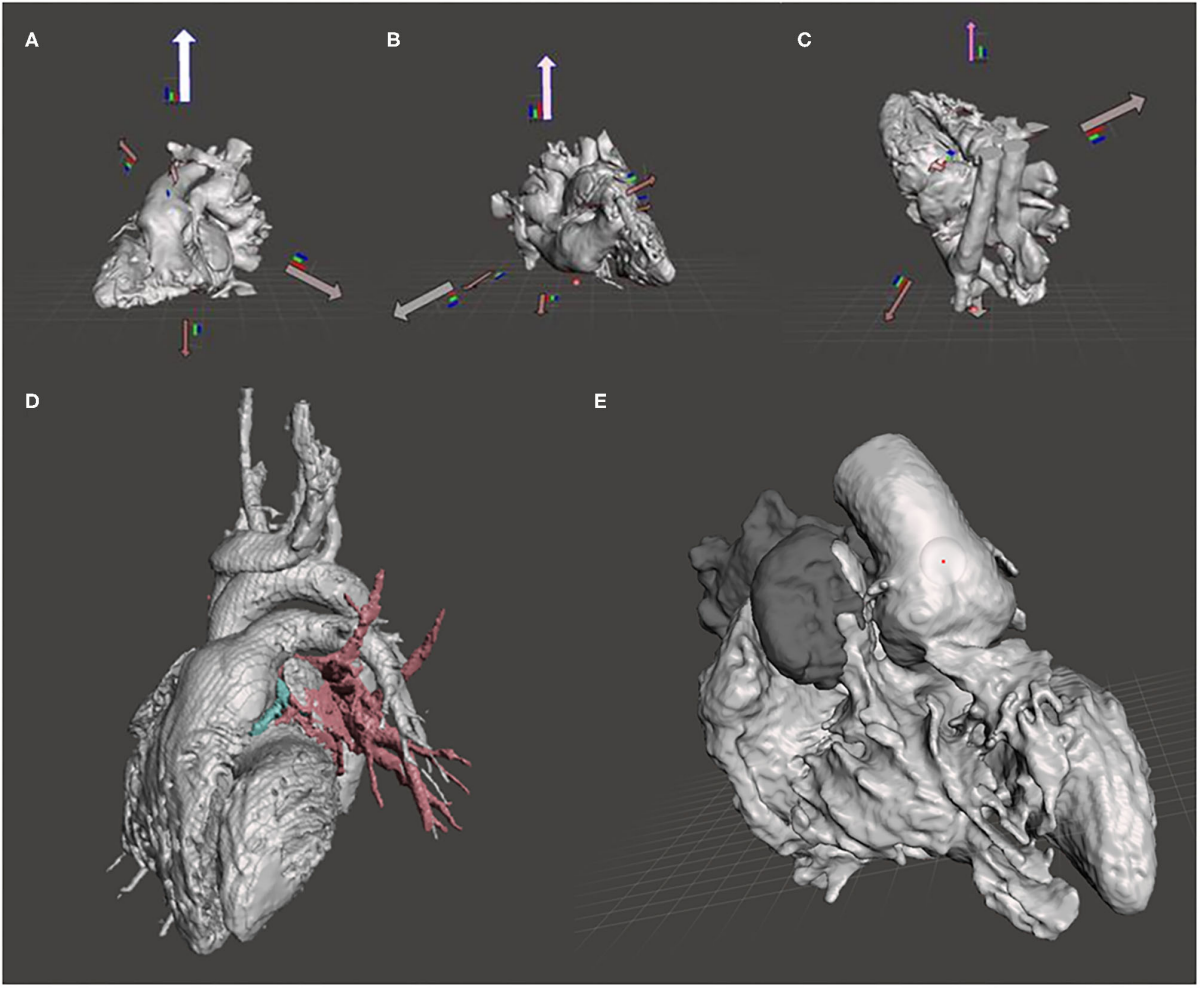
Tools and operations in Meshmixer. **(A–C)** Optimal printing positions of the heart model calculated by the orientation tool in Meshmixer. **(D)** Different anatomical structures segmented in ITK Snap. Blood pool in gray, pulmonary veins in red, and coronary in blue. **(E)** Smooth brush applied on the aorta surface.

We processed STL files in Meshmixer (Autodesk Inc., San Rafael, CA, USA). The meshes were analyzed with an automatic inspector tool to detect and repair errors (holes, isolated parts, and non-manifold surfaces). A 0.8-mm offset was applied to the meshes in order to keep the blood pool unmodified. Cantilever parts and slender supports were reduced or eliminated. The geometry was analyzed in every case to find the optimal orientation in the space prior to printing (Figure 2).

When the model was composed of several parts (i.e., aorta, pulmonary veins, inferior and superior cava veins, atriums, ventricles, etc.), all the meshes were merged using the combine tool in Meshmixer (Figure 2).

We used the sculpting module in Meshmixer to shape meshes with different brushes and parameters (Figure 2).

Once the 3D modeling operations were done, the accuracy of the model was checked. To fulfill this task, several key lengths in the model were compared with the information given by the cardiologist/ radiologist (6).

### Slicing

The open-source software Cura was used to generate the support structure for the model and to define all the parameters needed for the FDM printing. These parameters were as follows: type of material, Filaflex (Recreus, Elda, Spain); temperature of the nozzle, 230°C; size of the nozzle, 0.4 mm; speed of the printing head, 20 mm/s; layer thickness, 0.15 mm; wall thickness, 0.8 mm; and infill pattern, none.

**Figure 3:**
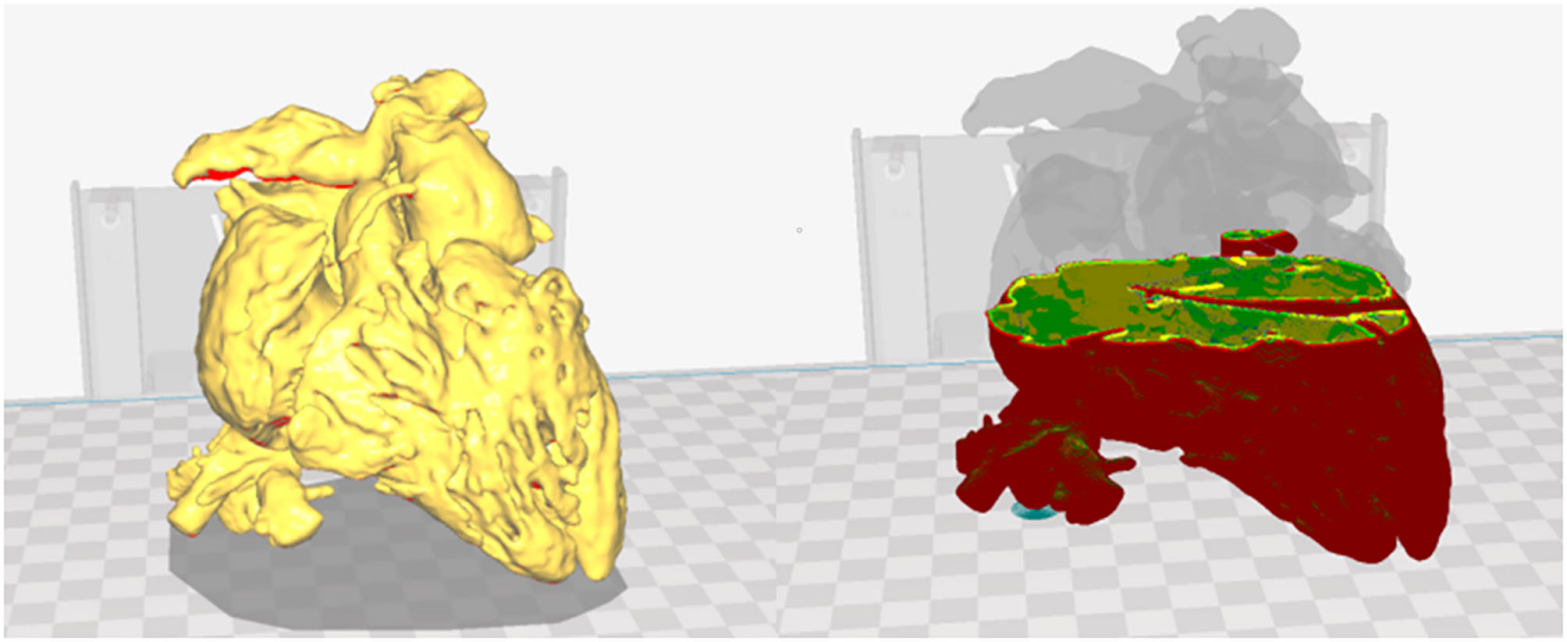
Printing parameters in Cura. Heart model visualization in Cura and virtual simulation of the printing process. Red: external layer; yellow: medium layer; and green: internal layer.

Before printing, the process was checked in a virtual simulation tool that showed all the layers and paths of the nozzle (Figure 3).

### 3D Printing

The BQ Witbox Fused Deposition Modeling (FDM) 3D printer was used to print all the heart models. This is a low-cost and open-source firmware and hardware printer capable to work with flexible filaments.

**Figure 4:**
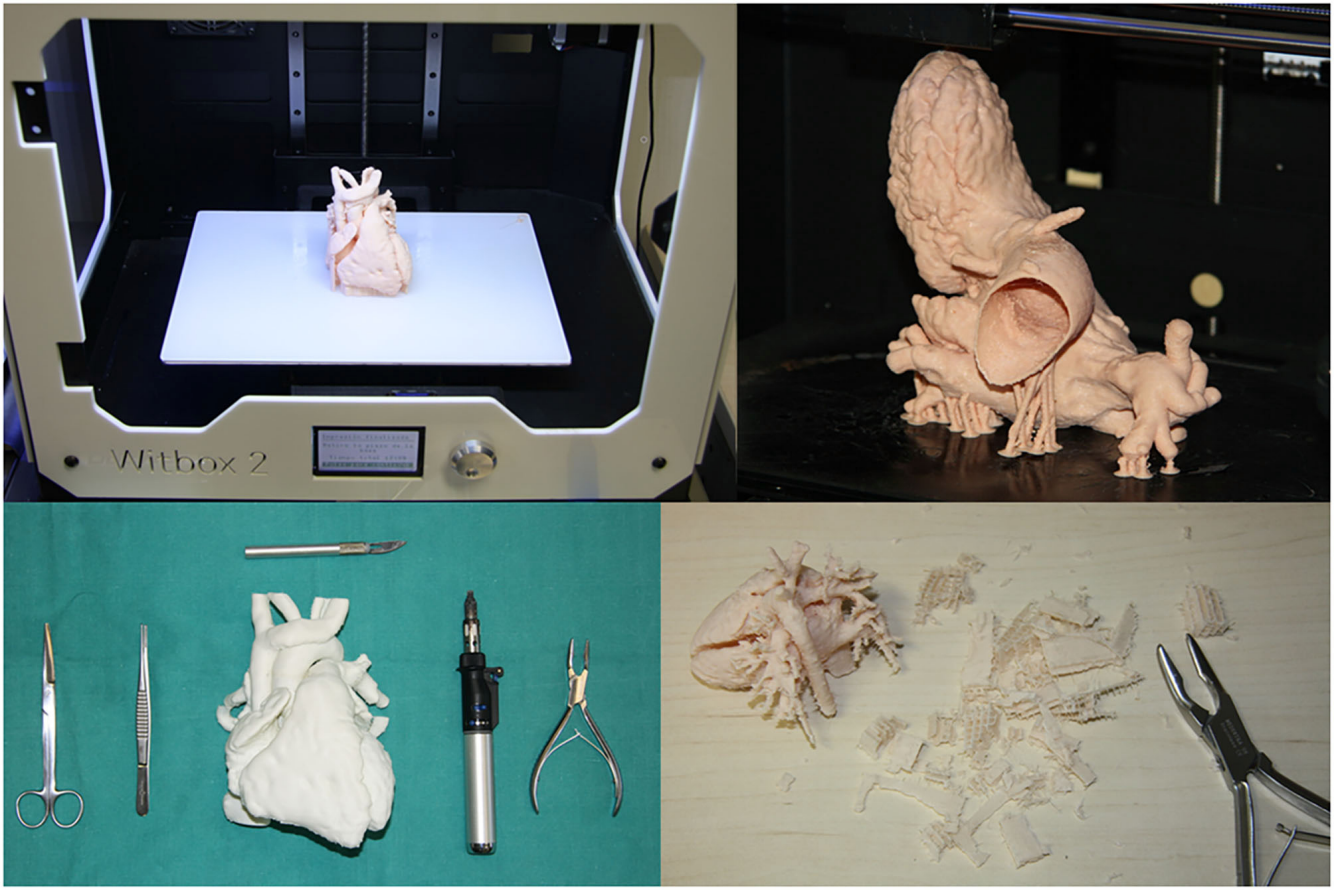
3D printing process and manual finishing operations. Heart model in BQ Witbox 2 printing plate (up left). Left heart model with supports (up right). Tools used for removing supports and repairing the model (scissors, tweezers, scalpel, gas blowtorch, and bone forceps).

G-Code files were generated in Cura (version 3.6.0). They were transferred to the printer to start the printing process. The maximum printing volume of the machine is 297 × 210 × 200 mm, enough to print an adult heart (Figure 4). The maximum resolution in the z-axis is 0.02 mm. The heart models were printed with 0.15-mm Z resolution.

Flexible filaments are highly adhesive on the printing plate, and there was no need for extra adhesion operations or devices such as lack spray, blue tape, or heated bed (Figure 4).

Models and supports were printed with the same material. Once printed, the support structures adhered to the piece were removed using tools such as scissors, tweezers, scalpel, gas blowtorch, and bone forceps (Figure 4).

The material used for printing was a thermoplastic polyether-polyurethane, low-temperature flexibility (Filaflex; Recreus Industries S.L., Elda, Spain). The most important features of Filaflex are as follows: printing temperature: 210–240°C; hardness, 82 Shore A; density, 1.12 g/cm^3^; tensile strength, 45 MPa; elongation at break: 650 %; and odorless and not soluble in water.

### Data Analysis

For each model, segmentation time (doctor), computer-aided design time (design expert), printing time, removing supports time (technician), overall amount of material needed, printing success, and cost were recorded. Printing success was defined as hearts correctly printed at first attempt.

Quantitative variables are presented as mean ± standard deviation.

The cost of the models is divided in different concepts: segmentation, computer-aided design, cleaning, printer amortization, and material.

The cost of the personnel has to be adapted to each country and legislation. In this study, the cost was estimated to be €35.59/h for doctors, €26.63/h for design experts, and €13.44/h for technicians.

The amortization of the printer has been calculated with an estimated duration of 4 years. Costs associated with the printer were as follows: 3D printer (€1,449.90, VAT included), 4 years maintenance plan (€200/years, VAT included). We have estimated 2,000 h a year of operating time. Filament cost was €52.03/kg.

## Results

### Design and Fabrication Process

The total number of heart models printed was 138: surgical planning 83 cases, interventional planning 48 cases, and interventional simulator seven cases. Images of the heart models numbers 1 to 42 are presented as Supplementary Figure 1a, heart models from 43 to 91 as Supplementary Figure 1b, and heart models from 92 to 138 as Supplementary Figure 1c.

The segmentation time was 63.82 ± 17.15 min, and the CAD time was 73.43 ± 18.9 min. The printing time was 13.35 ± 7.85 h, and it was directly related to the patient's heart size, which ranges from 2.08 to 24 h for children hearts and from 24 to 42.65 h for adult-size hearts. The cleaning time was 37.24 ± 8.42 min (Table 2).

**Table 2:** Summary of the heart model fabrication data.

	**Segmentation time (min)**	^*^CAD time (min)	**Printing time (h)**	**Cleaning time (min)**	**Material needed (g)**
Mean	62.62	73.28	13.02	36.77	60.55
Standard deviation	16.26	19.08	7.59	8.12	35.30
Max	115	125	42.65	58	186
Min	38	27	2.08	16	10

* *CAD, computer-aided design*.

All the data collected in this study are presented as Supplementary Table 1.

It was necessary to launch the printing process 251 times to have 138 successful 3D printed hearts.

The most important reasons for failure were clogging of the nozzle, support failure, and printing failure of overhanging structures.

The most important costs associated with the design and fabrication of the heart models were the segmentation process and CAD (Table 3). The rest of the factors associated with manufacturing barely had an impact on the final cost because throughout the process, low-cost materials and technologies were used.

**Table 3:** Mean cost of the design and fabrication process.

**Mean Cost (€/model)**	
Segmentation	€37.86
Computer-aided design	€32.59
Cleaning model	€8.34
Printer amortization (4 years, 2,000 h/year)	€3.67
Material	€3.2
Total	€85.66

### Clinical Applications of the 3D Printed Hearts

#### Surgical Planning

**Figure 5:**
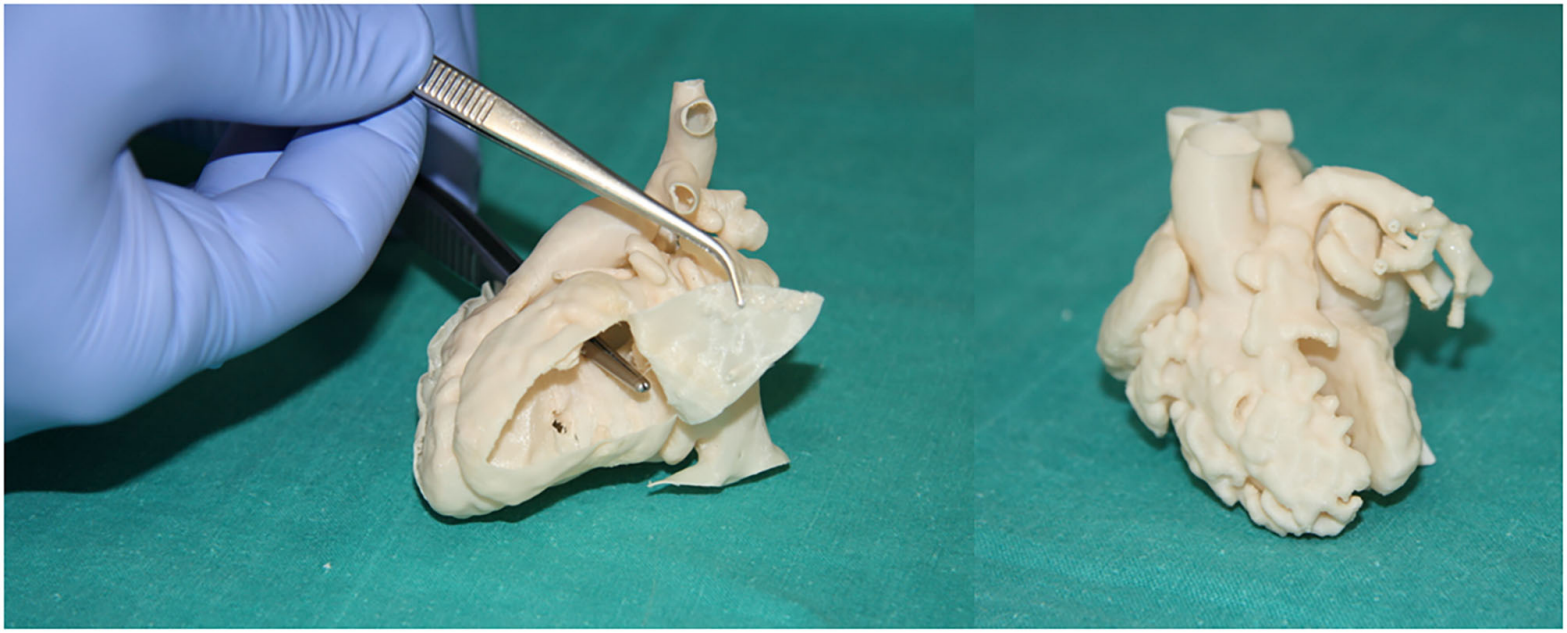
Examples of heart models for surgical and interventional planning. Double outlet right ventricle (Case 135), internal vision from the left ventricle (upper left). Double outlet right ventricle (Case 133), external view (upper right). Testing of the personalized catheterization simulator in real conditions. Two fluid inlets (superior and inferior cava vein) and two fluid outlets (aortic arch and descending aorta) (down left). Percutaneous pulmonary valve implantation in a patient with severe right ventricle dilatation. Interventional planning with 3D printed model and real intervention in the patient (down right).

A total of 83 out of 138 3D printed models were designed and manufactured for surgical planning. The congenital heart diseases treated were as follows: double outlet right ventricle (Figure 5), complex transposition of the great arteries, univentricular heart physiology, large ventricular septal defects, crisscross heart, left ventricle outflow tract obstruction, and heterotaxy syndrome, among others (2, 6, 29). As we demonstrated in a previous study (6), the surgical planning of these patients was modified in 47.5% of the cases compared to traditional surgical planning using only clinical information and medical imaging.

#### Interventional Procedures

**Figure 6:**
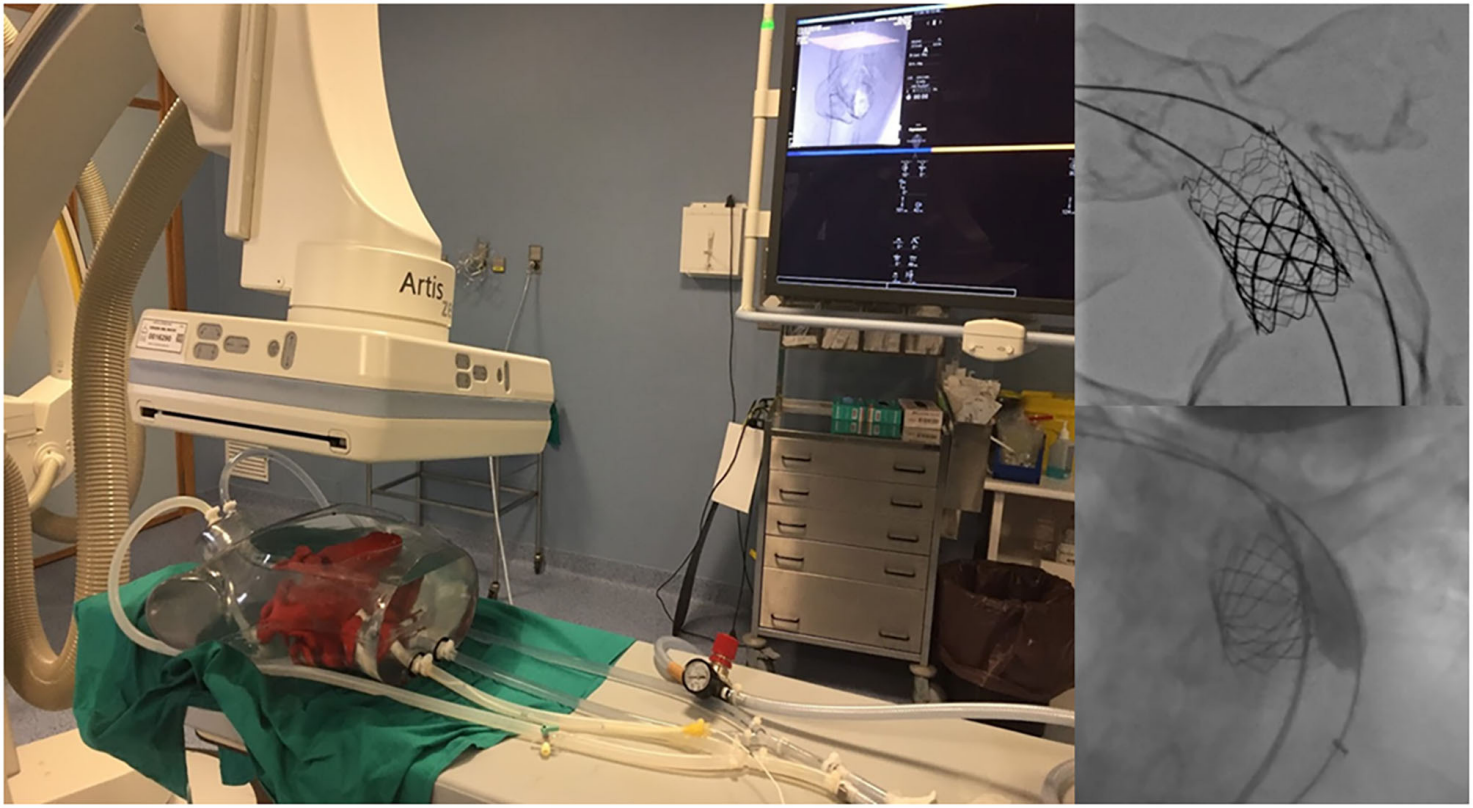
Catheterization applications. Testing of the personalized catheterization simulator in real conditions. Two fluid inlets (superior and inferior cava vein) and two fluid outlets (aortic arch and descending aorta) (left). Percutaneous pulmonary valve implantation in a patient with severe right ventricle dilatation. Interventional planning with 3D printed model (up right) and real intervention in the patient (down right).

Forty-eight heart models were used to plan catheterization procedures. There are several pathologies that can be planned or simulated using this technology. We have designed and manufactured models for aortic pathology, atrial septal defect closure, stent angioplasty of pulmonary venous baffle obstruction, percutaneous mitral annuloplasty, pulmonary valve implantation (Figure 6), and transcatheter aortic valve implantation, among others (2, 3, 30).

The mean segmentation cost was €37.86, the mean CAD cost was €32.59, the mean cleaning model cost was €8.34 €, the printer cost associated with each model was €3.67, and the mean 3D printing material cost was €3.2€. The total mean cost of the 3D printed hearts was €85.66 (Table 3). The amount of material needed for the hearts was 61.55 ± 36.03 g.

#### Education and Communication With Colleagues, Patients, and Their Relatives

Models were used to teach registrars and young clinicians and surgeons. Our team is currently working on a catheterization simulator where we expect to be able to perform different interventional procedures (Figure 6). The simulator is based on a pulsatile fluid pump and a network of plastic pipes emulating vascular structures (31).

The models were used in most cases for informed consent. Thanks to the use of the models, it was possible to improve the understanding of the pathology, as well as the surgical intervention to be performed and the possible risks involved.

A total of 13 cardiac surgeons and 30 pediatric cardiologists (from different hospitals and countries) were asked to give feedback on the models. The satisfaction rate on the use of the 3D models was quantified as 9.3 (surgeons) and 9.0 (pediatric cardiologists) out of 10, respectively; 67% of them agreed or strongly agreed that the model was useful to communicate with parents and patients (6).

## Discussion

### Key Findings of the Study

In this article, we present the most extensive series of 3D printed cardiac models published to date. We provide detailed information on how to implement a low-cost 3D printing laboratory for heart models.

In our study, the most important costs were those associated with the segmentation and computer design of the heart models (Table 3). The rest of the factors had little impact on the final cost because low-cost materials and technologies were used throughout the process.

The use of commercial software packages such as Mimics would have had an approximate economic impact of €10,000 per year.

Printing the heart models on a Stratasys Polyjet machine would have involved an equipment acquisition cost of over €100,000 and an average cost per heart of €196.19 in printing materials vs. € 3.2 with flexible filament in an FDM 3D printer (Supplementary Table 2).

### Impact of the Models on Clinical Management and Training

All the hearts that we have fabricated in the last 7 years have been used in clinical patients, and we have demonstrated a positive impact on congenital heart disease surgical decision process. Planning complex surgeries with a 3D printed heart can help medical teams to decide on the surgical approach. In our previous study, in 40% of the cases, the initial surgical plan was changed after reviewing the model (6).

We have studied and published the dimensional accuracy of the models, and we have found an excellent agreement between the dimensions of the 3D models and the medical images—CMR: mean difference −0.30 ± 0.67 mm, *t*-test *P* = 0.66; and CT images: mean difference −0.16 ± 0.85 mm, *t*-test *P* = 0.85 (6). The accuracy of the models allowed us to plan in detail specific interventions, and they have been essential in the development of new strategies such us percutaneous pulmonary valve implantation in large and dilated right ventricular outflow tracts (2).

Several authors have demonstrated the utility of 3D printing models to improve medical training (32). It is the scope of our future research to test how different 3D printed pathologic hearts can be used in the simulator in order to train clinicians to reproduce a wide range of catheterization procedures.

### Potential Short Comings From the Study

We believe that 3D printed models of the heart may become an essential tool for cardiologists and surgeons in interventional and surgical planning of complex cases. We hope that this publication facilitates the adoption of the technology in order to improve the quality of care of these patients.

### Future Considerations

New materials and 3D printing technologies are emerging every day. Automatic segmentation of the anatomy, 3D printing time reduction, human tissue-like materials, and biocompatible materials are the most important goals in which researchers are focusing nowadays. We are still far from being able to fabricate a complete and functional heart, but we are close to being able to produce patches and parts of the heart through tissue engineering.

## Conclusions

We have demonstrated that it is possible to print high-quality and affordable 3D printed cardiac models. These models have been useful to improve the quality of care of the patients who participated in the study.

## Data Availability Statement

The original contributions generated for the study are included in the article/Supplementary Material, further inquiries can be directed to the corresponding author/s.

## Ethics Statement

The studies involving human participants were reviewed and approved by Comité de Ética de Investigación Costa del Sol Luís Baro Rodríguez Alejandro Pérez Cabeza Francisco Rivas Ruiz Others. Written informed consent to participate in this study was provided by the participants' legal guardian/next of kin.

## Author Contributions

GG-C: funding acquisition, investigation, writing—original draft, and writing—review and editing. TG-C: funding acquisition, supervision, and writing—review and editing. JR-G and MV: research strategy and writing—review and editing. IV: funding acquisition, supervision, investigation, and writing—review and editing. All authors contributed to the article and approved the submitted version.

## Conflict of Interest

The authors declare that the research was conducted in the absence of any commercial or financial relationships that could be construed as a potential conflict of interest.
