# Supplementary figures and images for: Affordable Three-Dimensional Printed Heart Models
Source: Front Cardiovasc Med. 2021 Jun 4;8:642011. doi: 10.3389/fcvm.2021.642011 (PMC8211988; doi:10.3389/fcvm.2021.642011)

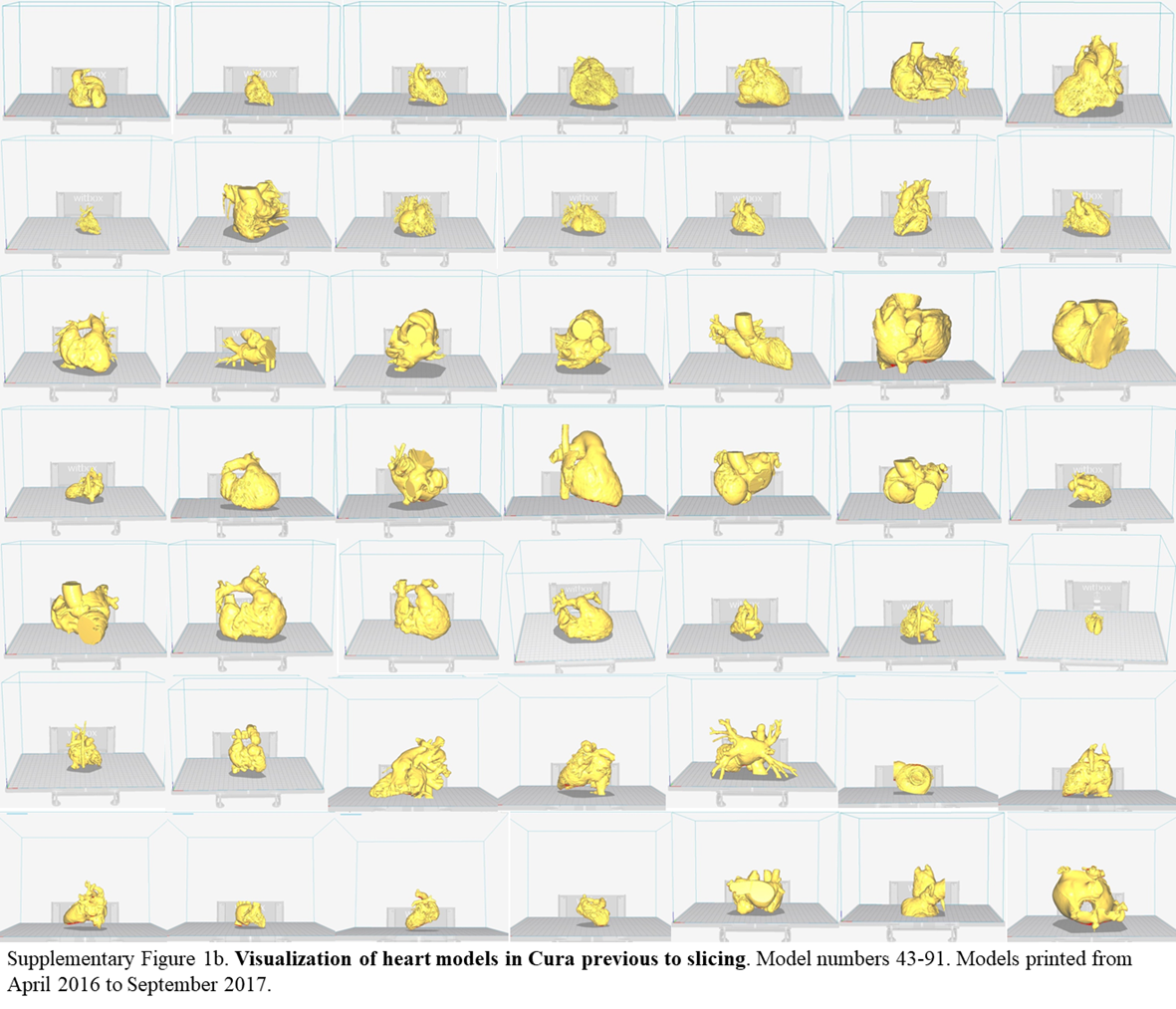

Supplement: Supplementary file 3 [file Image_1.TIF]

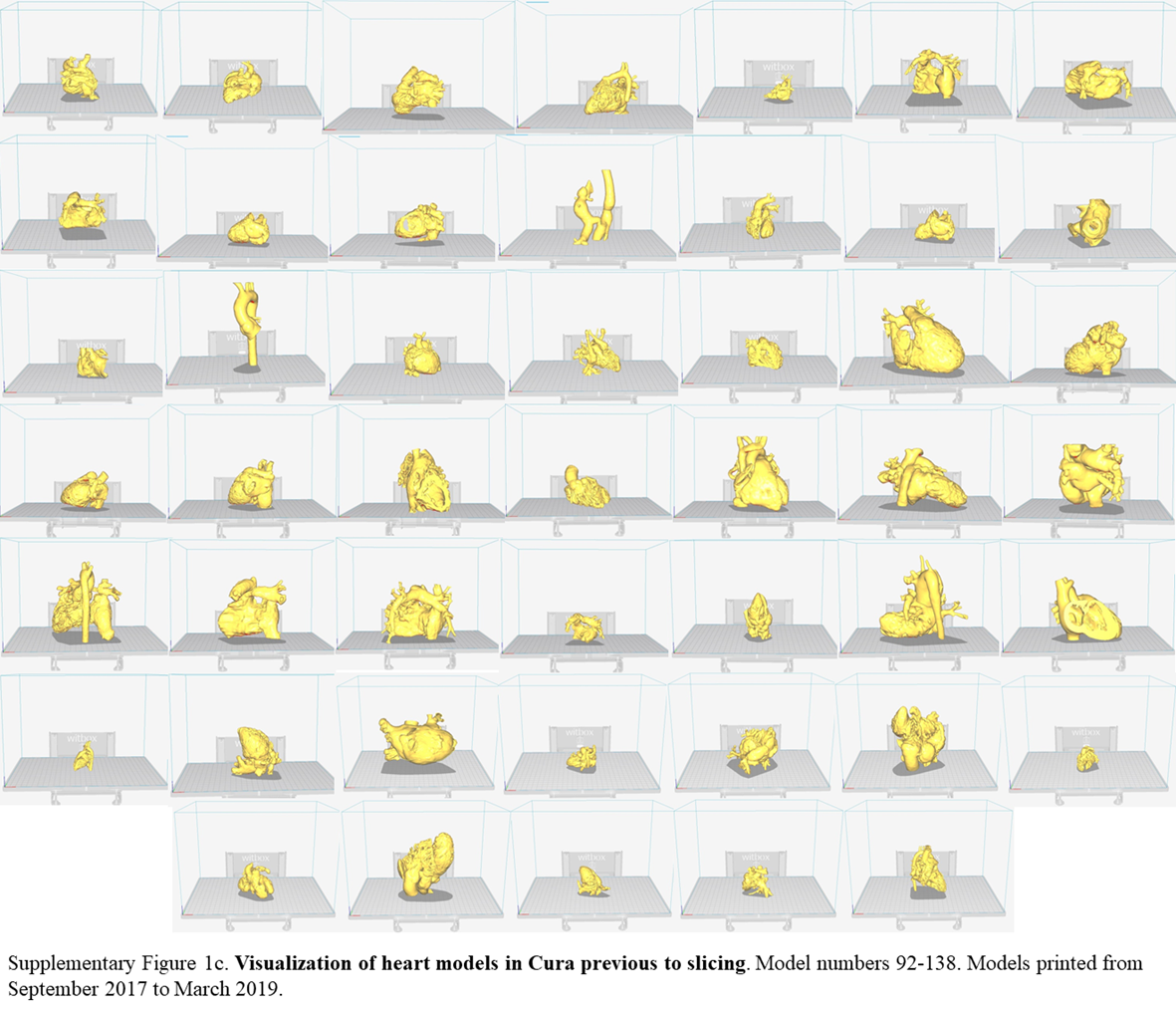

Supplement: Supplementary file 4 [file Image_2.TIF]

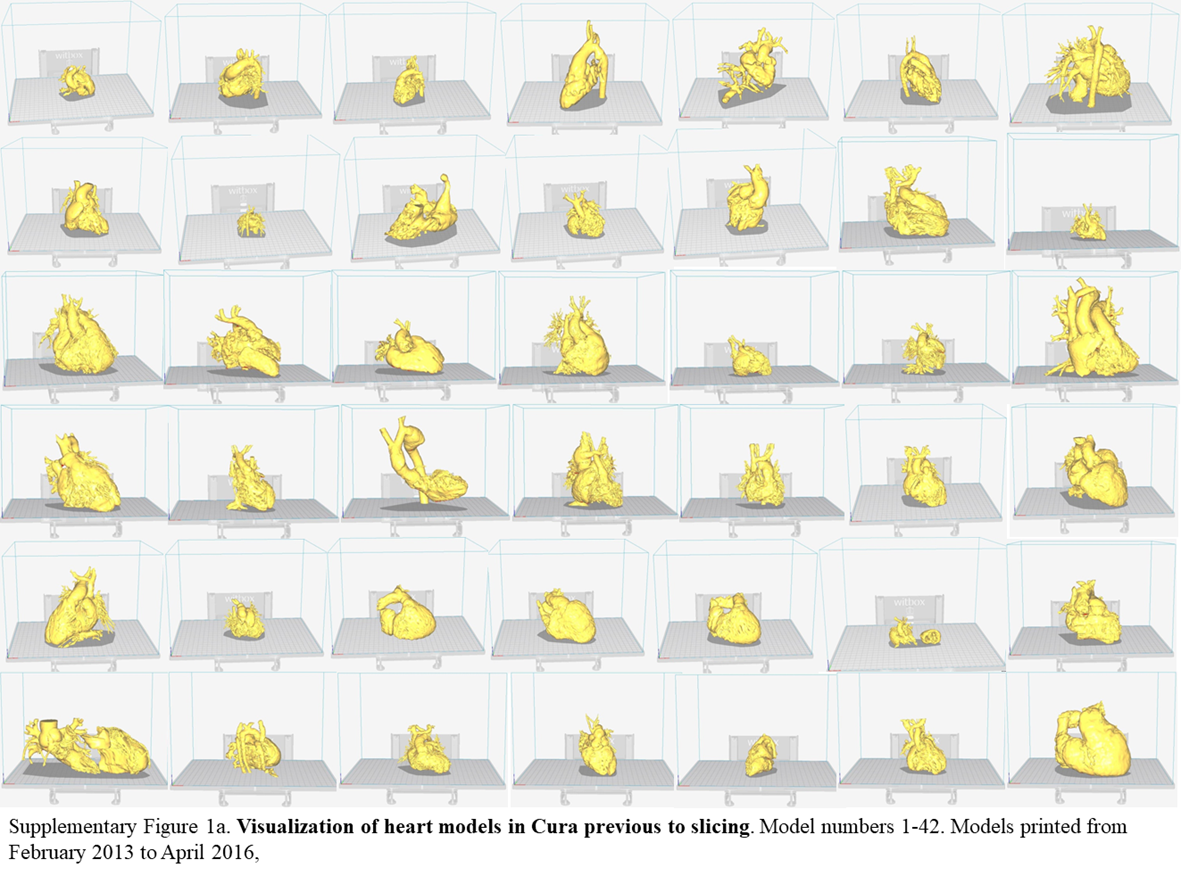

Supplement: Supplementary file 5 [file Image_3.TIF]
